# Association of ABCC2 and CDDP-Resistance in Two Sublines Resistant to CDDP Derived from a Human Nasopharyngeal Carcinoma Cell Line

**DOI:** 10.1155/2010/915046

**Published:** 2010-06-13

**Authors:** Si Ming Xie, Wei Yi Fang, Teng Fei Liu, Kai Tai Yao, Xue Yun Zhong

**Affiliations:** ^1^Cancer Research Institute, Southern Medical University, Guangzhou City, Guangdong Province 510515, China; ^2^Postdoctoral Station of Clinical Medicine, Medical College, Jinan University, Guangzhou City, Guangdong Province 510632, China; ^3^Pathology Department, Medical College, Jinan University, Guangzhou City, Guangdong Province 510632, China

## Abstract

Cisplatin (CDDP) is one of the most active drugs to treat nasopharyngeal carcinoma (NPC) patients. To further understand the mechanisms of CDDP-resistance in NPC, two CDDP-resistant sublines (CNE2-CDDP and CNE2-CDDP-5Fu) derived from parental NPC cell line CNE2 were established. It was found that at the IC50 level, the resistance of CNE2-CDDP and CNE2-CDDP-5Fu against CDDP was 2.63-fold and 5.35-fold stronger than that of parental CNE2, respectively. Of the four ABC transporters (ABCB1, ABCC1, ABCC2 and ABCG2) related to MDR, only ABCC2 was found to be elevated both in CDDP-resistant sublines, with ABCC2 located in nucleus of CNE2-CDDP-5Fu but not in CNE2-CDDP and parental CNE2. Further research showed that compared to untreated CNE2, the intracellular levels of CDDP were decreased by 2.03-fold in CNE2-CDDP and 2.78-fold in CNE2-CDDP-5Fu. After treatment with PSC833, a modulator of MDR associated transporters including ABCC2, the intracellular level of CDDP was increased in CDDP-resistant sublines, and the resistance to CDDP was partially reversed from 2.63-fold to 1.62-fold in CNE2-CDDP and from 5.35-fold to 4.62-fold in CNE2-CDDP-5Fu. These data indicate that ABCC2 may play an important role in NPC resistant to CDDP.

## 1. Introduction

Nasopharyngeal carcinoma (NPC) is a major malignant disease of the head and neck region and is endemic to Southeast Asia and Mediterranean basin, especially in Southern China [1, 2]. Since NPC originates from a hidden anatomical site and is more closely associated with advanced clinical stage, the results of conventional radiotherapy technique are unsatisfactory although it is a radiosensitive tumor. Therefore, chemotherapy treatment is a necessary ancillary method for these NPC patients [3, 4]. 

*cis*-Diamminedichloroplatinum (CDDP), also known as cisplatin, which is the most active and most frequently employed drugs, is often used to treat distant metastasis or advanced locoregional recurrence of NPC patients, and the chemotherapeutic regimen of CDDP plus 5-fluorouracil (5-Fu) is the main drug combination used in clinical trials [5, 6]. However, inherent and acquired resistance to the drug limits its applications in NPC chemotherapy. The mechanisms of resistance to CDDP are complicated. Of all these mechanisms, the one that is most commonly encountered in the laboratory is the increased efflux of abroad class of hydrophobic cytotoxic drugs that is mediated by one of a family of energy-dependent transporters, known as adenosine triphosphate binding cassette (ABC) transporters [7]. The ABC transporters have the capability to use energy to drive the transporters of various molecules across the cellular membrane and are confirmed to be associated with anticancer drug transporter [8].

Several members of the ABC transporter family can induce multidrug resistance (MDR). So far, tissue culture studies have consistently shown that the major mechanism of MDR in most cultured cancer cells involves ABCB1(also known as P-glycoprotein, P-gp, or MDR1), ABCC1 (also known as multidrug resistance associated-protein 1, MRP1), or ABCG2 [7]. ABCC2, also known as MRP2 or cMOAT, had been identified to confer cellular resistance of tumor cells to various anticancer drugs including CDDP [9]. Our previous study had confirmed that lentivirus-mediated RNAi silencing targeting ABCC2 might reverse the ABCC2-related drug resistance of NPC cell line CNE2 against CDDP [10]. 

To further illustrate the mechanism of CDDP-resistance, CDDP-resistant subline was established from parental NPC cell line CNE2. Given that the chemotherapeutic regimen of CDDP plus 5-Fu was widely used in clinic, CDDP-5Fu-resistant cell line derived from parental CNE2 was also established. PSC 833, a second-generation powerful MDR modulator [11], was utilized to reverse the CDDP-resistance in these cells. The modulation of ABCC2 expression was detected, and high performance liquid chromatograph (HPLC) was used to detect the intracellular accumulation of CDDP in these cells with or without PSC 833. 

## 2. Methods

### 2.1. Cell Lines and Cell Culture

The human NPC cell line CNE2 was grown in RPMI-1640 medium(Hyclone, Logan, UT) supplemented with 10% fetal calf serum (ExCell, Cranford, NJ) and 1% L-glutamine [12]. The CDDP-resistant and CDDP-5Fu-resistant sublines were generated by continuously culturing the drug-sensitive parental cell line CNE2 in medium containing large dose of CDDP (1 *μ*g/mL) and CDDP (1 *μ*g/mL) plus 5-Fu (1 *μ*g/mL) for over 1 year. To avoid an influence of CDDP or 5-Fu, all resistant sublines were cultured in drug–free medium for over 1 month before subsequent analysis. All Cells were cultured at 37°C in a humidified incubator with 5% CO2.

### 2.2. Cytotoxicity Assays by MTT

The cytotoxicity of CDDP to cells was determined using the MTT assay. Cells were seeded into 96-well plates at a density of 5 × 10^3^ cells per well. To investigate the effect of PSC 833, cells were incubated with or without 2 *μ*g/mL PSC 833 (Sandoz, Basel, Switzerland). Various gradient concentrations of CDDP (Qi Lu Pharmaceutical Factory, Jinan, China) ranging from 0.5 to 32 *μ*g/mL were added to each well 24 hours after seeding. Wells containing no drugs or PSC 833 were used to control cell viability, and wells containing no cells were used as a blank control. After 48 hours of incubation under normal culture condition, MTT was added at a final concentration of 5 mg/mL. Four h later, DMSO (Sigma, Louis, MO, USA.) was added to each well to dissolve the crystal with shaking horizontally for ten minutes [13]. The optical density (OD) value of 570 nm wavelength was measured by microplate reader (Bio Rad, Hercules, CA). The IC50 value, defined as the drug concentration required to reduce cell survival to 50% determined by the relative absorbance of MTT, was assessed by probit regression analysis in SPSS11.5 statistical software. The resistance index (RI) was calculated by dividing the IC50 of the CDDP-resistant sublines by that of parental cell line CNE2 [14]. All experiments were performed in triplicate.

### 2.3. Analysis of Cell Viability by MTT Assay

MTT assay was conducted to determine the cell proliferation. Cells were seeded into seven 96-well plates at a density of 1 × 10^3^ cells per well. Wells containing no cells were used as a blank control. A MTT assay was done daily from the second to the eighth day of incubation. The OD value of each well was measured as described above. Independent experiments were done in triplicate. Growth curves were drawn according to the OD values [15].

### 2.4. Analysis of ABCC2 mRNA Levels by Quantitative Real-Time PCR

Total RNA from all cells were extracted using Trizol reagent (Invitrogen, Carlsbad, CA) and reverse transcribed to cDNA using PrimeScript kit (Takara bio, Otsu, Japan) following the manufacturer's instructions. Real-time PCR was performed with MX3000P instrument (Stratagene, Cedar Creek, TX) using a commercially available master mix SYBR Premix Ex Taq kit (Takara bio, Otsu, Japan) containing Taq DNA polymerase and SYBR-Green I fluorescence dye. The primer sequences for target genes were as follows: ABCB1 forward, 5′-TAATGCGACAGG AGATAGG-3′, ABCB1 reverse, 5′-AAGAACAGGACTGATGGC-3′; ABCC1 forward, 5′-GAGGAAGGGAGTTCAGTCTT-3′, ABCC1 reverse, 5′-ACAAGACG AGCTGAATGAGT-3′; and ABCC2 forward, 5′-CTCACTTCAGCGAGACCG-3′, ABCC2 reverse, 5′-CCAGCCAGTTCAGGGTTT-3′; ABCG2 forward, 5′-ACCTG AAGGCATTTACTGAA-3′, ABCG2 reverse, 5′-TCTTTCCTTGCAGCTAAGAC-3′; The primer sequences for reference gene ACTB (also known as *β*-actin) were as follows: ACTB forward: 5′-CACCCAGCACAATGAAGAT-3′; ACTB reverse: 5′-CAAATAAAGC CATGCCAAT-3′. Cycling conditions were used as described previously [16]: 95°C for 10 minutes to activate DNA polymerase, followed by 45 cycles of 95°C for 15 seconds, 55°C for 20 seconds, and 72°C for 10 seconds. Specificity of amplification products was confirmed by melting curve analysis. Each sample was assayed in triplicate in independent reactions. The cycle threshold (Ct), which represents a positive PCR result, is defined as the cycle number at which a sample's fluorescence crossed the threshold automatically determined by the MX3000P system. The relative changes in gene expression were calculated with the 2^−ΔΔCt^ method, where ΔCt = Ct(target gene)—Ct(ACTB), and ΔΔCt = ΔCt (sample)—ΔCt (calibrator) [17, 18]. Here sample refers to CDDP-resistant and CDDP-5Fu-resistant CNE2 sublines, while calibrator refers to parental cell line CNE2.

### 2.5. Analysis of ABCC2 Protein Levels by Immunocytochemistry

Cells were seeded on slides, incubated under normal culture conditions for overnight, fixed in acetone for 30 minutes at 4°C, washed with PBS, and air-dried. After treated by endogenous biotin block system (MaiXin Biotechnology, Fuzhou, China), the slides were incubated in 3% H_2_O_2_ for 10 minutes to block endogenous peroxidase, and then in 10% normal rabbit serum for 10 minutes to block nonspecific antibody binding sites. When blocking was complete, the slides were then incubated with specific antibody against human ABCC2 ((Boster, Wuhan, China) in a 1 : 100 dilution in PBS at 4°C overnight. Subsequently, the slides were detected with the biotin-streptavidin- peroxidase system (Ultra Sensitive-SP kit; MaiXin Biotechnology, Fuzhou, China) at room temperature, using diaminobenzidine (MaiXin Biotechnology, Fuzhou, China) as color presentation (1–3 min). Counterstaining for nuclei was performed with hematoxylin. Negative controls were performed by substituting the specific antibody with PBS [19, 20]. 

### 2.6. Drug Accumulation Assays by High Performance Liquid Chromatograph

One day after cells were seeded in 25 cm^2^ flasks, CDDP (10 *μ*g/mL) was added to the flasks with or without PSC 833 (10 *μ*g/mL). Two h later, cells were harvested with 0.025% trypsin and 0.02% EDTA. Subsequently, they were counted by a haemocytometer, with total 10^6^ cells to be used. After washing by RPMI-1640 for three times, cell pellets were collected and resuspended in 0.3 ml distilled water, followed by freeze/thaw for 5 times to breakdown the cells. The mixtures were then centrifuged at 12,000 r.p.m for 30 minutes. Finally, 10 *μ*L of the supernatant containing CDDP content were analyzed by HPLC (series 1200, Agilent, Santa. Clara, CA, USA) [10, 21]. CDDP standard solutions ranging from 5 to 80 *μ*g/mL were used for preparation of calibration curve. Each sample was measured at least three times.

### 2.7. Statistic Analysis

All experiments were repeated at least three times. The data of quantitative RT-PCR, IC50, and intracellular accumulation of CDDP were expressed as mean ± SD value. Statistical analysis for these data was carried out by one-way ANOVA in statistical package SPSS 11.5. *P* < .05 was considered significant. 

## 3. Results

### 3.1. Resistance to CDDP is Enhanced in Drug-Resistant Sublines and Can Be Partially Reversed by PSC 833

**Figure 1:**
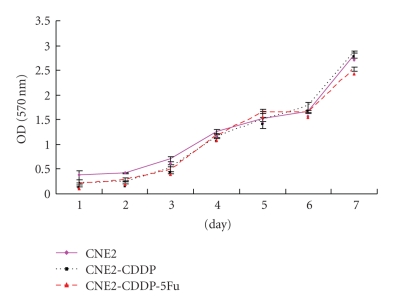
The growth curves of CDDP-resistant sublines and parental cell line CNE2. The cell viability was performed by MTT method for 7 continuously days.

The CDDP-resistant sublines, CNE2-CDDP, and CNE2-CDDP-5Fu, were generated after more than 1 year of culture. Cellular growth was determined by a continuous 7-day MTT assay, and growth curve was made according to the OD value alterations of MTT assay. No significant difference was found between the cellular growth of these cells (Figure 1). Table 1 summarized all the IC50 obtained testing CDDP. At the IC50 level, the resistance of CNE2-CDDP and CNE2-CDDP-5Fu against CDDP was 2.63-fold and 5.35-fold stronger than that of CNE2, respectively. In the parental cell line CNE2, PSC 833 was inactive, whereas in CNE2-CDDP and CNE2-CDDP-5Fu, the resistance against CDDP was reversed by PSC 833 from 2.63-fold to 1.62-fold and 5.35-fold to 4.62-fold, respectively. These data indicated that two CDDP-resistant sublines are established successfully, with more acquired CDDP-resistance in CNE2-CDDP-5Fu, and the CDDP-resistance can be partially reversed by PSC 833.

### 3.2. Of the ABC Transporters Related to MDR, ABCC2 mRNA is Highly Expressed in the Drug-Resistant Sublines

Several members of the ABC transporter family including ABCB1, ABCC1, ABCC2, and ABCG2 can induce MDR [7, 9]. Modulation of these ABC transporters was detected by relative real-time PCR. Of the four ABC transporters, ABCC2 was found to be upregulated both in CNE2-CDDP and CNE2-CDDP-5Fu (Table 2). Compared to untreated CNE2, the expression level of ABCC2 mRNA in CNE2-CDDP and CNE2-CDDP-5Fu was 2.46-fold and 4.29-fold increased, respectively. These data showed that in CDDP-resistant sublines, ABCC2 mRNA was overexpressed, with more expression level in CNE2-CDDP-5Fu. 

### 3.3. The Expression of ABCC2 Protein Is Enhanced in the Drug-Resistant Sublines

**Figure 2:**
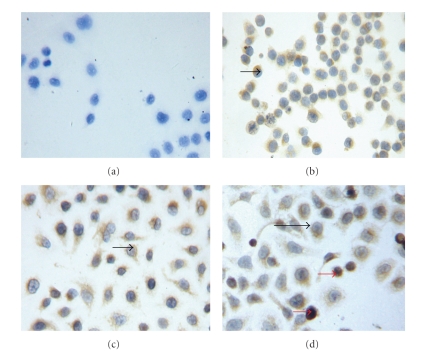
Immunocytochemical staining of ABCC2 expression in three cells (×200; SP method, DAB staining, and counterstaining with hematoxylin). (a) Blank control; (b) Parental cell line CNE2; (c) CDDP-resistant CNE2-CDDP cells; and (d) CDDP-5Fu-resistant CNE2-CDDP-5Fu cells. Black arrow: ABCC2 protein localized in cytoplasm; red arrow: ABCC2 protein localized in nucleus.

Since ABCC2 was found to be overexpressed both in CNE-CDDP and CNE2-CDDP-5Fu, and previous studies have shown that ABCC2 can be localized in the nuclear membrane of CDDP-resistant cells [22], immunocytochemistry staining method was used to detect the ABCC2 protein expression. As shown in Figure 2, ABCC2 protein was found to be localized in cytoplasm. The expression level of ABCC2 protein in CDDP-resistant sublines was stronger than that in parental CNE2. Interestingly, ABCC2 protein was also found to be localized in nucleus of CNE2-CDDP-5Fu cells. 

### 3.4. Intracellular Accumulation of CDDP in the Drug-Resistant Sublines Are Decreased and Can Be Increased by PSC 833

**Figure 3:**
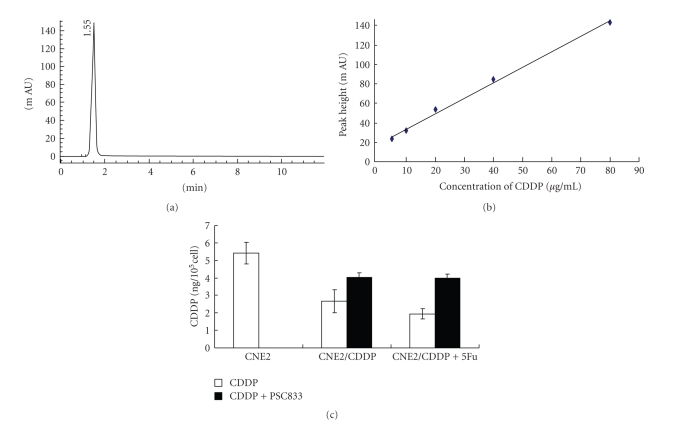
Intracellular accumulation of CDDP. (a) A typical chromatogram for total analysis of CDDP using HPLC determination. (b) Calibration curve for gradient concentration of CDDP within the range of 5–80 *μ*g/ml. A typical linear relationship (*R* ^2^ = 0.9965) was found between peak height and concentration of cisplatin. (c) The cellular accumulation of CDDP in parental CNE2 and resistant sublines. The concentration of CDDP was determined according to the calibration curve of CDDP. CNE2 resistant sublines were treated with or without PSC 833. *P < .05 versus CNE2.

ABC transporters have the capability to use energy to drive the transporters of various molecules across the cellular membrane [8]. As a measure of the functional activity of ABCC2 which was overexpressed in CDDP-resistant sublines, the detection of intracellular accumulation of CDDP was carried out by HPLC. As shown in Figure 3(a), a symmetrical peak for typical chromatograms of CDDP was found, and retention time for CDDP was 1.55 minutes. Figure 3(b) showed a typical linear relationship (*R* ^2^ = 0.9965) between peak height value and gradient concentration of CDDP. The equation obtained from this calibration curve is *y* = 1.59*x* + 17.92, where *y* stands for the peak height value of CDDP and *x* stands for the concentration of CDDP. Thereby the intracellular concentration of CDDP for each sample was analyzed (shown in Figure 3(c)). Compared to untreated CNE2, the intracellular level of CDDP was decreased by 2.03-fold in CNE2-CDDP and 2.78-fold in CNE2-CDDP-5Fu. In contrast, after treatment with PSC 833, the intracellular level of CDDP was decreased from 2.03-fold to 1.33-fold in CNE2-CDDP and from 2.78-fold to 1.35-fold in CNE2-CDDP-5Fu, which indicate that the intracellular accumulation of CDDP in CNE2-CDDP and CNE2-CDDP-5Fu was increased after PSC 833 treatment. No significant modulation was found in CNE2. These data suggest that the capacity to drive CDDP out of cells in CDDP-resistant sublines is enhanced and can be partially reversed by PSC 833.

## 4. Discussion

Chemotherapy with CDDP is widely used to treat NPC patients. Primary and secondary resistance to CDDP are major limitations to their use in cancer chemotherapy. Improved understanding of the biologic mechanisms leading to CDDP resistance will provide molecular targets for therapeutic intervention and may facilitate prediction of response and therewith the basis for individually tailored therapy. 

To further understand the mechanisms of CDDP-resistance, two CDDP-resistant sublines, CNE2-CDDP and CNE2-CDDP-5Fu, derived from parental NPC cell line CNE2, were established. The resistance was found to be stable for over 1 month in drug-free medium. No difference was found between the cellular growth of these cells. The resistance against CDDP, compared to untreated CNE2, was 2.63-fold for CNE2-CDDP and 5.35-fold for CNE2-CDDP-5Fu, respectively. These data suggest that the CDDP-resistant sublines were established successfully, with more acquired resistance against CDDP in CNE2-CDDP-5Fu than that in CNE2-CDDP. 

After the CDDP-resistant sublines were generated, the mRNA levels of the four members of ABC transporter family, ABCB1, ABCC1, ABCC2, and ABCG2, which were found to be associated with MDR [7], were detected by real-time PCR. Only ABCC2 was found to be up-regulated both in CNE2-CDDP and CNE2-CDDP-5Fu and was confirmed by immunocytochemichal staining. ABCC2 had been shown to be a unidirectional ATP-driven export pump localized mainly in the canalicular membrane of hepatocytes [23]. In vitro data suggest that ABCC2 might act as an organic anion pump [24, 25]. On the other hand, the expression of ABCC2 was found to be elevated in a number of cell lines selected for CDDP resistance [9, 26]. In addition, our previous study had demonstrated that the specific RNAi targeting ABCC2 led to greater cytotoxicity of CDDP to CNE2 [10]. These data indicated that ABCC2 is related to CDDP-resistance. In this study, compared to CNE2-CDDP, increased expression of ABCC2 was observed in CNE2-CDDP-5Fu, which was found to be shown more acquired CDDP-resistance than CNE2-CDDP. Our results imply that ABCC2 may play an important role in the CDDP-resistance of NPC cell line CNE2.

Previous studies confirmed that ABCC2 was localized in the nuclear membrane of CDDP-resistant cells, and nuclear membranous localization of ABCC2 correlated with resistance against CDDP in ovarian carcinoma cells [22, 27]. Furthermore, it had been reported that after treatment with RNAi targeting ABCC2, decreased nuclear membranous ABCC2 protein expression in the CDDP-resistant cancer cell lines was observed [28]. In this study, ABCC2 protein was also found to be localized in the nucleus of the more CDDP-resistant subline CNE2-CDDP-5Fu, but not in CNE2-CDDP and parental CNE2. The cellular target of CDDP has long been believed to be DNA, for it has been shown to bind DNA and cause DNA duplex to bend and unwind. Thus, ABCC2 may protect the nucleus from formation of platinum-DNA adducts by driving CDDP out of the cytoplasm and nucleus. 

To further confirm that CDDP can be driven out of cells by ABCC2, HPLC, which is an effective method to detect the intracellular accumulation of CDDP, was applied. HPLC was a rapid, economic, and validated way to determine the accumulation of CDDP in plasma, cancer cell, and tumor samples [29]. Our results showed that the intracellular accumulation of CDDP in CNE2-CDDP and CNE2-CDDP-5Fu was decreased compared to CNE2. Since in the four ABC transporters (ABCB1, ABCC1, ABCC2, and ABCG2) related to drug resistance, only ABCC2 was found to be up-regulated both in CNE2-CDDP and CNE2-CDDP-5Fu, and previous study had demonstrated that after treatment with RNAi targeting ABCC2, the cellular accumulation of CDDP in CNE2 was increased [28], it was inferred that probably ABCC2 has the capacity to drive CDDP out of the cytoplasm of CDDP-resistant sublines. Furthermore, compared to CNE2-CDDP, less intracellular accumulation of CDDP was found in CNE2-CDDP-5Fu. It was noted that ABCC2 was found to be localized in nucleus of CNE2-CDDP-5Fu. These data indicated that may be nuclear localized ABCC2 has the capacity to drive CDDP out of the nucleus of CNE2-CDDP-5Fu, which results in more acquired resistance to CDDP. 

The MDR-associated transporters inspired a wide search for compounds that would not be cytotoxic themselves but would inhibit MDR transporters. The first of these drugs to be studied was PSC 833, a nonimmunosuppressive cyclosporine D derivative and less toxic compared to cyclosporine A, and also known to inhibit ABCC1 and ABCC2 [11, 30, 31]. In our research, after treatment with PSC 833, the intracellular accumulation of CDDP in subline CNE2-CDDP and CNE2-CDDP-5Fu was increased, with increased cytotoxicity of CDDP. Given that of the four ABC transporters, only ABCC2 was found to be up-regulated both in CNE2-CDDP and CNE2-CDDP-5Fu, and PSC 833 was a modulator of MDR-associated transporters including ABCC2, the role of ABCC2 in driving CDDP out of cytoplasm may be interfered by PSC 833, which further imply that ABCC2 may play an important role in CDDP-resistance.

In our research, ABCC2 was found to be localized in nucleus of CNE2-CDDP-5Fu, but not in CNE2-CDDP. Moreover, less intracellular accumulation of CDDP and more acquired resistance against CDDP were found in CNE2-CDDP-5Fu compared to CNE2-CDDP. These results suggest that the combination of CDDP and 5-Fu may result in the nuclear localization of ABCC2, thus CDDP may be driven out of nucleus and more resistance against CDDP was acquired. The regimen of CDDP plus 5-Fu chemotherapy is widely used to treat NPC patients. Our results imply that perhaps such a regimen will result in greater cytotoxicity to tumor cells, yet it will make the remaining tumor cells more resistant to CDDP, probably mediated by ABCC2, and finally lead to poor prognosis. The relationship between the expression of ABCC2 in NPC patients and CDDP-resistance in clinic should be further studied. 

## 5. Conclusion

Taken together, derived from parental NPC cell line CNE2, two CDDP-resistant sublines (CNE2-CDDP and CNE2-CDDP-5Fu) were developed, hence a good cell model to study the mechanisms of CDDP-resistance was established. We demonstrated that the expression of ABCC2 was both elevated in CNE2-CDDP-5Fu and CNE2-CDDP, and ABCC2 protein was found to be localized in nucleus of CNE2-CDDP-5Fu, but not in CNE2-CDDP and parental CNE2. Furthermore, less intracellular accumulation of CDDP in CDDP-resistant sublines compared to parental CNE2 was found. Therewith, more acquired CDDP-resistance was found in CDDP-resistant sublines compared to CNE2. Of these cell lines, the most resistance against CDDP was found in CNE2-CDDP-5Fu, with ABCC2 localized in nucleus and the least intracellular accumulation of CDDP. The intracellular accumulation of CDDP and CDDP-resistance can be partially reversed by PSC 833, a modulator of MDR-associated transporters including ABCC2. These data suggest that ABCC2 may play an important role in NPC resistant to CDDP, indicating that ABCC2 may be taken as one of the targets to overcome CDDP-resistance in NPC. 

## Figures and Tables

**Table 1:** Modulation of resistance against CDDP with or without PSC 833.

Cell lines	IC50 (RI)	
	CDDP	CDDP plus PSC 833
CNE2	0.90 ± 0.14 (1)	0.89 ± 0.25 (1)
CNE2-CDDP	2.37 ± 0.37 (2.63)*	1.44 ± 0.10 (1.62)**
CNE2-CDDP-5Fu	4.82 ± 0.84 (5.35)*	4.11 ± 0.25 (4.62)**

RI: resistance index (x-fold) compared to parental CNE2

*(**): *P* < .05 versus CNE2.

**Table 2:** Relative quantification of four ABC transporters.

ABC transporters	CNE2	CNE2-CDDP		CNE2-CDDP-5Fu	
	ΔCt	ΔCt	Fold change (2^−ΔΔCt^)	ΔCt	Fold change (2^−ΔΔCt^)
ABCB1	22.6 ± 0.3	22.8 ± 0.5	0.87	22.2 ± 0.5	1.32
ABCC1	12.8 ± 0.2	13.3 ± 0.2	0.71	14.1 ± 0.1	0.41
ABCC2	10.9 ± 0.1	9.6 ± 0.1*	2.46	8.8 ± 0.1*	4.29
ABCG2	16.2 ± 0.2	16.0 ± 0.6	1.15	14.8 ± 0.5*	2.64

*P < .05 versus CNE2.

## References

[1] Wu J, Xiao X, Zhao P (2006). Minicircle-IFN*γ* induces antiproliferative and antitumoral effects in human nasopharyngeal carcinoma. *Clinical Cancer Research*.

[2] Her C (2001). Nasopharyngeal cancer and the southeast Asian patient. *American Family Physician*.

[3] Ahmad A, Stefani S (1986). Distant metastases of nasopharyngeal carcinoma: a study of 256 male patients. *Journal of Surgical Oncology*.

[4] Lee AWM, Lau WH, Tung SY (2005). Preliminary results of a randomized study on therapeutic gain by concurrent chemotherapy for regionally-advanced nasopharyngeal carcinoma: NPC-9901 trial by the Hong Kong Nasopharyngeal Cancer Study Group. *Journal of Clinical Oncology*.

[5] Ngan RKC, Yiu HHY, Lau WH (2002). Combination gemcitabine and cisplatin chemotherapy for metastatic or recurrent nasopharyngeal carcinoma: report of a phase II study. *Annals of Oncology*.

[6] Dimery IW, Hong WK (1993). Overview of combined modality therapies for head and neck cancer. *Journal of the National Cancer Institute*.

[7] Szakács G, Paterson JK, Ludwig JA, Booth-Genthe C, Gottesman MM (2006). Targeting multidrug resistance in cancer. *Nature Reviews Drug Discovery*.

[8] Dean M, Rzhetsky A, Allikmets R (2001). The human ATP-binding cassette (ABC) transporter superfamily. *Genome Research*.

[9] Taniguchi K, Wada M, Kohno K (1996). A human canalicular multispecific organic anion transporter (cMOAT) gene is overexpressed in cisplatin-resistant human cancer cell lines with decreased drug accumulation. *Cancer Research*.

[10] Xie SM, Fang WY, Liu Z (2008). Lentivirus-mediated RNAi silencing targeting ABCC2 increasing the sensitivity of a human nasopharyngeal carcinoma cell line against cisplatin. *Journal of Translational Medicine*.

[11] Thomas H, Coley HM (2003). Overcoming multidrug resistance in cancer: an update on the clinical strategy of inhibiting P-glycoprotein. *Cancer Control*.

[12] Zhou N-N, Zhu X-F, Zhou J-M (2004). 2-methoxyestradiol induces cell cycle arrest and apoptosis of nasopharyngeal carcinoma cells. *Acta Pharmacologica Sinica*.

[13] Wu X, Fan W, Xu S, Zhou Y (2003). Sensitization to the cytotoxicity of cisplatin by transfection with nucleotide excision repair gene xeroderma pigmentosun group A antisense RNA in human lung adenocarcinoma cells. *Clinical Cancer Research*.

[14] Ishida Y, Ohtsu T, Hamada H (1989). Multidrug resistance in cultured human leukemia and lymphoma cell lines detected by a monoclonal antibody, MRK16. *Japanese Journal of Cancer Research*.

[15] Zheng X, Shen G, Yang X, Liu W (2007). Most C6 cells are cancer stem cells: evidence from clonal and population analyses. *Cancer Research*.

[16] Seyhan AA, Vlassov AV, Ilves H (2005). Complete, gene-specific siRNA libraries: production and expression in mammalian cells. *RNA*.

[17] Livak KJ, Schmittgen TD (2001). Analysis of relative gene expression data using real-time quantitative PCR and the 2^−ΔΔ*C*_T_^ method. *Methods*.

[18] Smith JL, Rangaraj K, Simpson R (2004). Quantitative analysis of the expression of ACAT genes in human tissues by real-time PCR. *Journal of Lipid Research*.

[19] Krag DN, Kusminsky R, Manna E (2005). The detection of isolated tumor cells in bone marrow comparing bright-field immunocytochemistry and multicolor immunofluorescence. *Annals of Surgical Oncology*.

[20] Gao L, Lu C, Xu C, Tao Y, Cong B, Ni X (2008). Differential regulation of prostaglandin production mediated by corticotropin-releasing hormone receptor type 1 and type 2 in cultured human placental trophoblasts. *Endocrinology*.

[21] Lanjwani SN, Zhu R, Khuhawar MY, Ding Z (2006). High performance liquid chromatographic determination of platinum in blood and urine samples of cancer patients after administration of cisplatin drug using solvent extraction and N,N′-bis(salicylidene)-1,2-propanediamine as complexation reagent. *Journal of Pharmaceutical and Biomedical Analysis*.

[22] Surowiak P, Materna V, Kaplenko I (2006). Nuclear membraneous localization of ABCC2(MRP2,cMOAT) in ovarian carcinoma cells can correlate with resistance to platinum-containing drugs and clinical outcome. *Proceedings of the American Association for Cancer Research*.

[23] Keppler D, Leier I, Jedlitschky G (1997). Transport of glutathione conjugates and glucuronides by the multidrug resistance proteins MRP1 and MRP2. *Biological Chemistry*.

[24] Evers R, Kool M, van Deemter L (1998). Drug export activity of the human canalicular multispecific organic anion transporter in polarized kidney MDCK cells expressing cMOAT (MRP2) cDNA. *The Journal of Clinical Investigation*.

[25] Borst P, Kool M, Evers R (1997). Do cMOAT (MRP2), other MRP homologues, and LRP play a role in MDR?. *Seminars in Cancer Biology*.

[26] Kool M, de Haas M, Scheffer GL (1997). Analysis of expression of cMOAT (*MRP2*), MRP3, MRP4, and MRP5, homologues of the multidrug resistance-associated protein gene (MRP1), in human cancer cell lines. *Cancer Research*.

[27] Guminski AD, Balleine RL, Chiew Y-E (2006). MRP2 (ABCC2) and cisplatin sensitivity in hepatocytes and human ovarian carcinoma. *Gynecologic Oncology*.

[28] Materna V, Stege A, Surowiak P, Priebsch A, Lage H (2006). RNA interference-triggered reversal of ABCC2-dependent cisplatin resistance in human cancer cells. *Biochemical and Biophysical Research Communications*.

[29] Lopez-Flores A, Jurado R, Garcia-Lopez P (2005). A high-performance liquid chromatographic assay for determination of cisplatin in plasma, cancer cell, and tumor samples. *Journal of Pharmacological and Toxicological Methods*.

[30] Liscovitch M, Lavie Y (2002). Cancer multidrug resistance: a review of recent drug discovery research. *IDrugs*.

[31] Böhme M, Büchler M, Müller M, Keppler D (1993). Differential inhibition by cyclosporins of primary-active ATP-dependent transporters in the hepatocyte canalicular membrane. *FEBS Letters*.

